# French‐style genetics v. 2.0: The “e‐CohortE” project

**DOI:** 10.1111/cge.13595

**Published:** 2019-07-11

**Authors:** Henri‐Corto Stoeklé, Marc Bollet, Aurélie Cobat, Philippe Charlier, Oudy Ch. Bloch, Jérôme Flatot, Clément Draghi, Valérie Tolyan, Christian Hervé, Pierre Desvaux, Laurent Uzan, Michaël Grynberg, Alexandre Alcaïs, Alain Tolédano, Guillaume Vogt

**Affiliations:** ^1^ Neglected Human Genetics Laboratory CEA Evry France; ^2^ Centre National de Recherche en Génomique Humaine (CNRGH), Direction de la Recherche Fondamentale CEA, Institut de Biologie François Jacob, Université Paris Saclay Evry France; ^3^ Institut Sapiens Paris France; ^4^ Institut Rafaël, Maison de l'après cancer Levallois‐Perret France; ^5^ Institut de Radiothérapie et de Radiochirurgie, H Hartmann Levallois‐Perret France; ^6^ Sorbonne Paris Cité, Imagine Institute Paris Descartes University Paris France; ^7^ Laboratory of Human Genetics of Infectious Diseases INSERM UMR 1163, Necker Branch Paris France; ^8^ Département de la Recherche et de l'Enseignement Musée du Quai Branly ‐ Jacques Chirac Paris France; ^9^ UVSQ (Laboratoire DANTE ‐ EA 4498) Montigny‐le‐Bretonneux France; ^10^ Attorney Paris France; ^11^ Sociéte Flatot Paris France; ^12^ International Academy of Ethics, Medicine and Public Health Paris Descartes University Paris France; ^13^ Department of Urology Cochin hospital Paris France; ^14^ Paris Descartes University Sorbonne Paris Cité Paris France; ^15^ Institut Coeur Effort Santé Paris France; ^16^ Service de Médecine de la Reproduction & Préservation de la Fertilité Hôpital Antoine Béclère Clamart France; ^17^ French National Reference Center for Primary Immune Deficiencies (CEREDIH) Necker‐Enfants Malades University Hospital, Assistance Publique‐Hôpitaux de Paris Paris France; ^18^ Neglected Human Genetics Laboratory INSERM, Université Paris Descartes Paris France

## Abstract

In the digital age, a genetics cohort has become much more than a simple means of determining the cause of a disease. Two‐sided markets, of which 23andMe, Ancestry DNA and MyHeritage are the best known, have showed this perfectly over the last few years: a cohort has become a means of producing massive amounts of data for medical, scientific and commercial exploitation, and for genetic use in particular. French law does not currently allow these foreign private companies to develop on French national territory and also forbids the creation of similar entities in France. However, at least in theory, this same law does not preclude the creation of new types of cohorts in France inspired by the success of two‐sided markets but retaining features specific to the French healthcare management system. We propose an optimal solution for France, for genomic studies associated with multi‐subject questionnaires, still purely theoretical for the moment: the development, with no need for any change in the law, of France's own version of “Genetics v.2.0”: “e‐CohortE.”

## Graphical abstract

*It is the overall organization and functioning of this project*. In the digital age, a genetics cohort has become much more than a simple means of determining the cause of a disease. Two‐sided markets, of which 23andMe, Ancestry DNA and MyHeritage are the best known, have showed this perfectly over the last few years: a cohort has become a means of producing massive amounts of data for medical, scientific and commercial exploitation, and for genetic use in particular. French law does not currently allow these foreign private companies to develop on French national territory and also forbids the creation of similar entities in France. However, at least in theory, this same law does not preclude the creation of new types of cohorts in France inspired by the success of two‐sided markets but retaining features specific to the French healthcare management system. We propose an optimal solution for France, for genomic studies associated with multi‐subject questionnaires, still purely theoretical for the moment: the development, with no need for any change in the law, of France's own version of “Genetics v.2.0”: “e‐CohortE.”

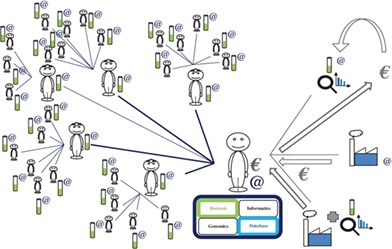

## 1 BACKGROUND

French genetic research still has a relatively “classical,” if not archaic view of the nature of a cohort.^1^ As highlighted by a recent expert opinion from the French national consultative committee for ethics (CCNE),* apart from being rather small, French cohorts are subject to a number of other problems: data dispersion, gaps in interoperability and insufficient sharing and coordination of resources. Two national platforms are being set up to deal with these difficulties: the French national plan for genetic data, MFG‐2025 (*Plan France Médecine Génomique* 2025†) and the Health data hub.‡ However, the real effectiveness of these platforms remains, at this stage highly relative. The problem is mostly due to the organization and functioning of cohorts in France.

Typically, a cohort is defined as a means of studying the health of different individuals over time. In this framework, we talk about cohorts of patients or of individuals from the general population, but cohort studies have historically had a restricted scope in any case. However, the new two‐sided markets,^2^, ^3^, ^4^ such as 23andMe,§ Ancestry DNA** and MyHeritage,†† have clearly showed that, in the digital era, in genetics, limiting the scope of research has become a scientific and economic limitation. Indeed, there is a clear advantage to studying everything possible in the general population, without prior assumptions, to identify genetic features more easily and, often, serendipitously.

These two‐sided markets have created new cohorts designed neither to identify the cause nor determine the natural course of a specific disease. Their purpose is to generate massive amounts of data, including genetic data in particular, that can be transformed into different types of information, some useful and others suitable for valorization.^4^, ^5^ These cohorts have been restructured and reorganized so as to function as an industrial means of producing data and information relating to genetics, connected or unconnected to health. It is clear that this new cohort model has proved itself both scientifically and economically.^6^, ^7^ 23andMe illustrates this perfectly, with its cohort of several million individuals, with and without particular diseases. Thus, even though these private companies are banned in France, they have already published dozens of scientific publications and have a capital of several billion dollars,^8^ amassed in less than 15 years.

Whether we like it or not, this success, however, debatable, raises questions about the efficacy of current French cohorts, for genetics studies at least.^1^ In the face of the results of 23andMe, current French cohorts appear to be obsolete and in need of profound reorganization to render them more effective and more competitive globally. France now has only two options. The first is to change the law, which, in theory, prohibits these two‐sided markets from developing in France, and, in practice, prevents similar entities from being created in France.‡‡ However, this choice entails a risk of offering these already established two‐sided markets a monopoly on our soil, given the huge lead they already have over any French competition. The second option is to leave the law unchanged and to create cohorts inspired by these two‐sided markets, but with beneficial characteristics more compatible with the current French system.^9^, ^10^

Like these two‐sided markets, these cohorts would be multi‐themed, with dynamic consent and virtual questionnaires completed with the use of new information and communication technologies, to allow participant autonomy and to make it possible to develop different research themes over time, from cancers to physical or behavioral traits, for example, if desired. However, these cohorts would not be two‐sided markets, but would correspond to a specific research protocol (“*recherche impliquant la personne humaine (RIPH)”* §§ in French, or research involving humans).

The goal of this RIPH would be genomic studies associated with multi‐subject questionnaires. This would initially make it possible to determine the genetic factors easiest to identify (ie, monogenic factors) without prior assumptions, because an infinite number of questions could be posed. This would accelerate genome mapping and the discovery of more complex (ie, polygenic) genetic factors.

Within the framework of an RIPH, participation would be free and would be supported by a public or private academic structure (hospital, institute, university etc.). Unlike two‐sided markets, they would receive data of a uniformly high quality (ancestry and true scientifically validated predispositions) and any medical predispositions detected would be managed free‐of‐charge by the French national health insurance system, which is accessible to all. In these conditions, French participants, who generally trust the French state to ensure the security of their personal data, might prefer these RIPHs over foreign two‐sided markets.

This is the only solution that would enable France to deal effectively with these two‐sided markets and their genetic tests, which are otherwise destined to spread throughout France, with or without legal restrictions. We present here an optimal solution, which we have called e‐CohortE (e‐CE), providing an overview of its organization and global functioning.

### 1.1 e‐CohortE

**Figure 1:**
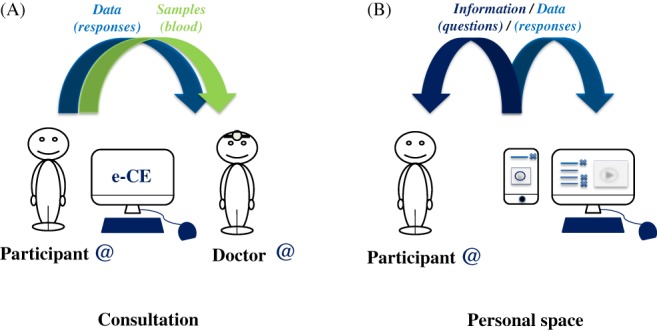
Modeling of interactions between the participants and doctors. A, Each participant is informed, by a doctor, of the objectives of the study and the means of achieving its ends. The participant provides electronic dynamic consent and answers a series of questions in the presence of the doctor. B, The participant receives an identifier for connection to the computer interface of the study at home or elsewhere, to enable him/her to answer other questions online and to receive more detailed information [Colour figure can be viewed at http://wileyonlinelibrary.com]

In this system, recruitment begins with the presentation, on a digital support, of the information notice, followed by the signing of the electronic consent form by the participant during a consultation with one of the investigating doctors for the protocol. The information notice would, of course, explain the ultimate aim: “genomic studies associated with multi‐subject questionnaires.” This consent is, therefore, virtual (Figure 1A).^11^ It is established between the participant (who may or may not be a patient) and the investigating doctor, via a computer, a computer tablet, or a smartphone. The investigating doctor reads and explains aloud the entire content of the information notice and the virtual consent form to the participant, in accordance with French law, before obtaining the informed consent of the participant.

The information notice should be as explicit as possible, and the investigating doctor must be present when the participant signs the electronic consent form. The participants then belong to the cohort for a research protocol in human genetics and may, if they wish, obtain a printed version of their consent and the information notice. Following signature of the consent form, an observation notebook, also in electronic form, is completed by the investigating doctor, together with the entry questionnaire. This entry questionnaire contains a short set of original questions common to the entire cohort. Of course, investigating doctors, given their specialty or interest, can choose to ask the participant additional validated questions.

When giving express consent, the participant may, of course, authorize access to his or her shared medical dossier (DMP, for “*dossier médical partagé*” in French),*** which, depending on the structure, may contain an authorization to access the national health data system (système national des données de santé, SNDS),††† itself consisting of the national information system for the various regimes of the national health insurance system (système national d'Information inter‐régimes de l'Assurance maladie, SNIIRAM)‡‡‡; data from hospitals and other healthcare establishments (under the program for the medicalization of information systems—programmede médicalisation des systèmes d'Information, PMSI§§§); statistical data for causes of death (Basede données sure les Causes Médicales de Décès en France, BCMD)**** and, in the near future, data relating to disabled individuals.

The participants may answer all or only some of the questions, in the knowledge that they do not need to justify the absence of a response to a particular question and that this absence will not invalidate research relating to other questions that they have answered. The participants may, either at home or elsewhere, at any time, answer the other questions to which they did not respond at the time of recruitment or that the doctor did not necessarily ask them, given his or her specialty. This situation is made possible by the electronic nature of the research protocol and the availability of new information and communication technologies.

**Figure 2:**
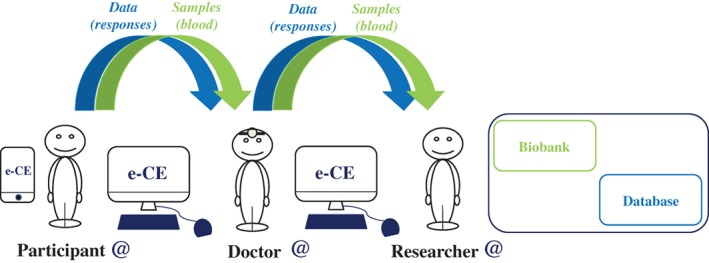
Modeling of interactions between participants, doctors and researchers (1). At the end of the consultation, following the signing of dynamic electronic consent and the issuing of an identifier for logging on to the interface, a biological sample is collected from the participant. This sample is rendered anonymous and sent to the coordinating researcher for storage in the biobank. The responses to questionnaires, also rendered anonymous, are stored directly in the database [Colour figure can be viewed at http://wileyonlinelibrary.com]

**Figure 3:**
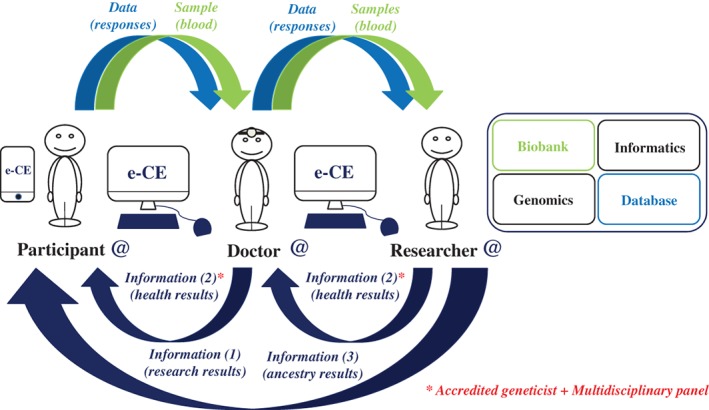
Modeling of interactions between participants, doctors and researchers (2). The genetic data obtained from a genomics platform are analyzed and combined with the responses to the questionnaires present in the database via a computer platform, which returns the data directly to the participant without passing by the doctor. The data delivered are the overall results of the research (information (1)). Some information relating to the health of the participant (information (2)) may also be returned to the participant, if the participant so wishes. However, this information is subject to prior expert evaluation and validation by an accredited geneticist and a multidisciplinary committee before its transmission to the doctor and then to the participant during a consultation. Other information relating to the ancestry of the participant may be delivered directly to the participant online (information (3)) [Colour figure can be viewed at http://wileyonlinelibrary.com]

At the end of this first consultation, the participants receive an identifier (a “login” name) and a password, with all the necessary security precautions, to allow them to connect to the study website via the Internet on a computer or via a smartphone application. Thus, wherever they happen to be, the participants can access their personalized secure accounts, in which they can find their consent forms, information notices (enriched content: videos) and questionnaires. All the questions, those that the participant has already answered and new questions added to the website, are accessible to the participants online, allowing them to answer other questions relating to the study, but also to reconsider the responses to the questions posed by the investigating doctor at the time of recruitment (Figure 1B). Progress in the study and the people performing the study are clearly visible to the participants, who can, of course, withdraw from the protocol or modify their responses to questionnaires or to particular questions at any time.

e‐CE can thus be classified as category 2 research on humans (according to the Jardé Law), involving the analysis of genetic data and totally free‐of‐charge for the participant. It thus constitutes a research protocol in human genetics, making it possible to sample and use diverse biological materials. Following signature of the electronic consent form, the investigating doctor or qualified staff can take the first biological sample (in this case a blood sample) for high‐quality DNA extraction (Figure 1A). The diverse samples are stored in a biobank specific to the protocol. It is therefore essential to add to such a protocol the possibility and explanation of additional visits for the participant, with his or her agreement, for the collection of other samples (saliva, skin biopsy, stools, sweat, tears, etc.), depending on the scientific questions addressed and according to the expected or unexpected nature of the results of future research collaborations.

e‐CE would make it possible to create an online computer interface (website) “connecting” participants in research studies, doctors, investigating researchers and study coordinators for the storage and use of data (responses to questions, test results, database) and biological samples (Figure 2). Each question would be traceable: provenance (public or private laboratory), state of progress (date of the study) and bibliography (state‐of‐the‐art relating to the question, legitimacy of the study). Following this information, each time that a participant answers a question, he or she will see the response statistics (percentage of responses for the cohort) and, later, he or she will have access to the overall results of the study (publications, talks, meetings and vulgarization of the study). Thus, for each response given, the participants will be informed, over time (via messages or a smartphone application), of the progress of the study concerned and whether additional questions are available to refine the study. This is, indeed, one of the strengths of such an interface and, above all, of such a protocol: if the subjects so wish, they have direct access to the results of each study linked to the questions posed, and much more (researcher → participant) (Figure 3).

According to the needs and desires of the centers, in addition to the genome study relating to the questions posed, the e‐CE could eventually provide participants with information concerning about a hundred genetic characteristics, already scientifically validated by previous studies and explained via the participant's personalized account (Figure 3). These genetic data, with the possible exception of ancestry data, could be generated by next‐generation sequencing and individually checked by Sanger sequencing, which would differentiate this type of research from the genotyping testing services that can be bought abroad. These genetic characteristics would be susceptibility factors for physical traits, tolerances (alcohol, caffeine, drugs) or risk factors for disease (ie, actionable disease††††). Again, according to the needs and desires of the two parties (participants and researchers), other results, information relating to the participant's ancestry (ethnic origin), could be supplied directly to the participants online (researcher → participant), in accordance with French and European regulations (Figure 3). The participant can, of course, at any time, accept or refuse to receive such information (in the initial consent and subsequently online if the participant changes his/her mind). Nothing in French law clearly bans the delivery of such results within the framework of an RIPH protocol.

Indeed, the French national data protection agency comission nationale de l'Informatique et des libertés (the CNIL) defines health data as data of a personal nature relating to health, including “information obtained in tests or examinations of part of the body or of a bodily substance, including genetic data.” Based on this definition and Article L1122‐1‐1 of the French Public Health Code, during an RIPH, “The person whose participation is sought is informed of his or her right to receive the information concerning his or her health held by the investigator, during or at the end of the study.”

It should be noted that these health‐related data are not medical diagnoses, but could constitute information potentially useful to doctors who could use them to decide whether to perform appropriate genetic tests or to obtain validation of the results by Sanger sequencing at an accredited center, resulting in a recognized medical diagnosis, with a proposed care/prevention pathway. Thus, these divulgences of information requested by participants, particularly those relating to health, could be regulated by subjecting them to prior expert assessment and validation by an accredited geneticist and a multidisciplinary committee before their transmission to the investigating doctor and then the participant and, potentially his or her relations (in accordance with French law) during a consultation (researcher → doctor → participant) (Figure 3). The participant can agree or refuse to receive such information at any time (in the initial consent and subsequently online if the participant changes his/her mind). The investigating doctor should contact the patient to discuss the information, before the participant views it directly, to explain what the results mean. If the investigating doctor prefers this type of information to be divulged to his or her patient by another intermediary, he or she should indicate an appropriate care pathway.

The investigating doctors can connect to the interface and have access only to the data and information relating to their own patients in the protocol. They are located at the heart of exchanges with participants, forming an essential link of trust for e‐CE, and helping participants. The doctor‐participant relationship and the opinion of the ethics committee (CPP or committee for the protection of persons) concerning the performance of such a study are the only two elements that should really be taken into account. An interpretation of our laws by a state regulatory body with its own doctrine not representing the views of the patients would not be appropriate in this context. In this way, a protocol respecting the participants and our laws in France is different from two‐sided markets.

Depending on the needs and preferences of the doctor responsible for treatment, doctors can communicate in at least five different ways with their patients: (a) physically, via a consultation, (b) visually or orally, by videoconference or telephone, (c) in writing, via their private or professional messaging service (available via the website), (d) via a smartphone application or (e) via the forum. Researchers have access to all the available information, but, importantly, not the personal identifiers of the participants (Figure 3). The researchers can make use of the data for the entire cohort and can study it through the questions posed in the questionnaires. The researchers can also communicate via messages with the participants who answered their questions. Similarly, the participants can address questions to the researchers of the research protocols in which they participate. Thus, researchers can increase the attractiveness of their studies by providing richer information online.

The discussion forum makes it possible to adapt the content as a function of feedback from the participants. The participants thus have the possibility, under cover of a pseudonym, to share information about the protocol or questionnaires or to contact the investigating doctors or researchers of the protocol. It should be possible to adapt e‐CE to their expectations, within the framework of French law, through this participatory research. The questions and suggestions for improvement of the participants should push the system towards more transparent research.

### 1.2 Perspectives

**Figure 4:**
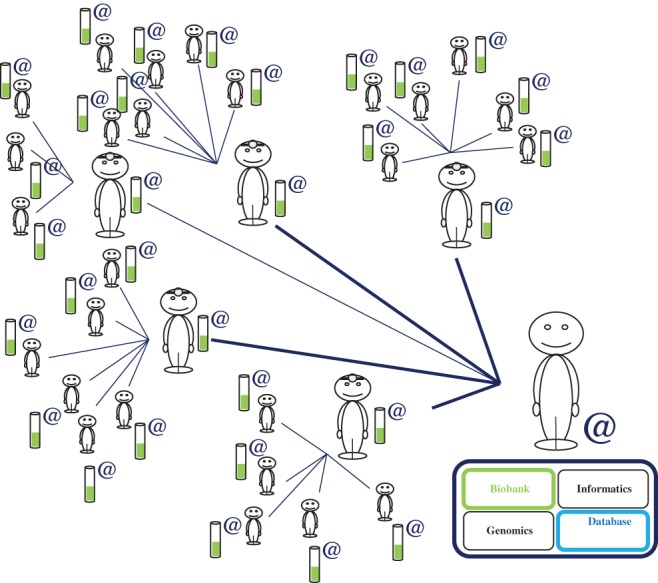
Modeling of the interactions between participants, doctors and researchers. Densification of the participant‐doctor‐researcher “network.” As the cohort is both connected and dynamic, the objective is to increase, progressively, the numbers of participants and doctors over time and space (avoiding limitation to a single hospital or town, for example) [Colour figure can be viewed at http://wileyonlinelibrary.com]

**Figure 5:**
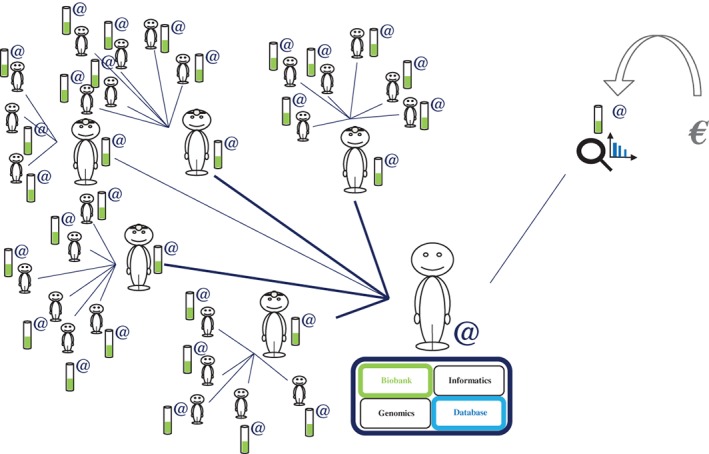
Modeling of interactions between participants, doctors and researchers, plus external researchers. Once the cohort has been set up, another researcher from a different academic laboratory with the necessary funding can collaborate with e‐CE and pose another question (possibly after a simple amendment to the protocol if this question was not already present) [Colour figure can be viewed at http://wileyonlinelibrary.com]

**Figure 6:**
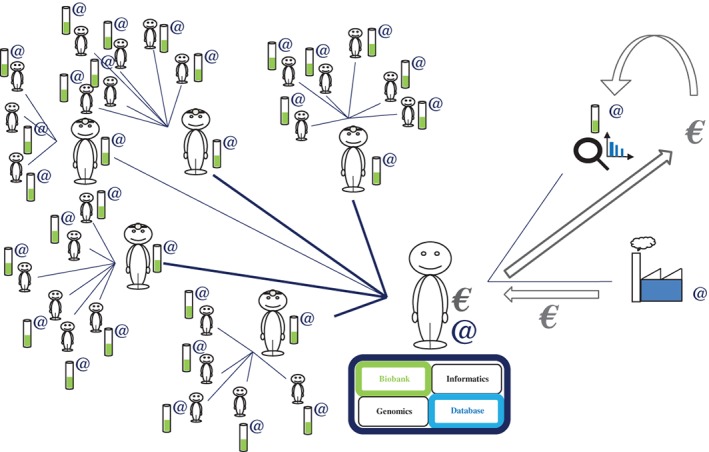
Modeling of interactions between participants, doctors and researchers, plus external researchers and companies. A private company can also pose “its” questions to “our” cohort, in the same way as an academic laboratory would do. On each occasion, the participants are informed of the source of the question [Colour figure can be viewed at http://wileyonlinelibrary.com]

Once the cohort (biobank, database) and its network of participants, doctors, investigators, researchers and coordinators have been constituted via the RIPH (anything from 1 to an infinite number of participants [Figure 4]), another researcher from a different academic laboratory with his or her own research funding can collaborate with the e‐CE and pose “his or her” own question to “our” cohort (Figure 5). Indeed, this would represent both a saving of time and of funding for the researcher concerned. The researcher would thus have at his or her disposition a ready‐built infrastructure that is already functioning, and would therefore need to find funding only for the generation of the genetic data of interest at cost price (in the case of collaboration with a genomics platform), provided that the participants respond to the new question online and thus give express consent via the interface. Evidently, if the question posed by the researcher/doctor concerns a particular population unlikely to be represented in the cohort, e‐CE could open a new recruitment center with the doctor concerned, on an ad hoc basis linked to the question, with a simple amendment to the protocol approved by the CPP (necessitating a delay of 0 to 2 months). The researcher would therefore be able to obtain the appropriate population after a simple amendment rather than the writing of a new genetic research protocol and its approval (delay of 6 to 18 months) with or without the storage of genetic data.

e‐CE will not close its doors to pharmaceutical and biotech companies. This will also differentiate it from certain public cohort protocols clearly stipulating that no sharing or exchange of data with the private sector is permitted. A private company (pharmaceutical or biotech company) can, therefore, pose “its” question to “our” cohort, in the same manner as an academic laboratory (again, subject to consent and to the maintenance of participant confidentiality, thanks to the traceability of questions) (Figure 6). The participant sees simply that the question comes from an industrial source, and it is the responsibility of the company concerned to show that its question is well‐founded in the enriched information note placed online on the interface for this theme. Furthermore, the participant will know that, in the framework of e‐CE, any financial surplus resulting from this exchange will be used for the performance of other genetic analyses in the cohort, increasing the density of the network, improving the infrastructure of the cohort (machines, website, moderators, doctors online) or offering funding (for genetic analyses, the constitution or analyses of questionnaires) for other research questions from academic laboratories needing to reach a large connected cohort in record time.

**Figure 7:**
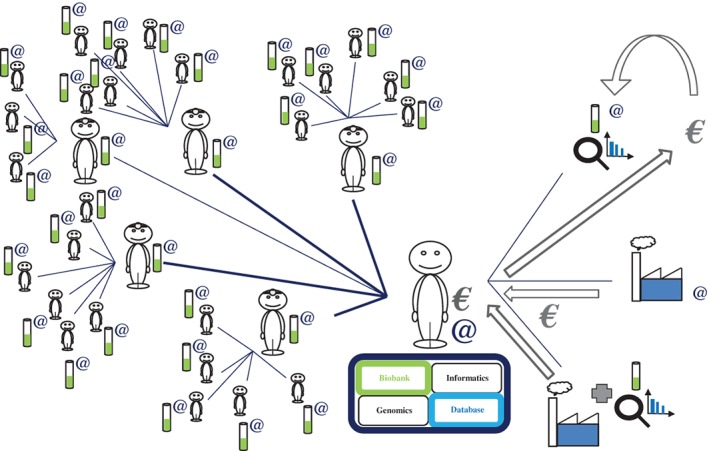
Modeling of interactions between participants, doctors and researchers plus external researchers and companies. In the case of collaboration in an interventional study with a private or public sector partner, or of a collaboration between public and private sector partners, the participants of the cohort with profiles corresponding to that sought are contacted via the computer interface. The participants are free to participate in this new study, by signing a new consent form independent of that for the main protocol. Thus, e‐CE makes it possible to obtain information or to investigate the possibility of recruitment for other studies and for other sponsors [Colour figure can be viewed at http://wileyonlinelibrary.com]

In the case of collaboration for interventional research (Jardé Law category 1) with a private organization, or a public/private‐sector collaboration, the participants of the cohort corresponding to the profile sought are informed via the computer interface (Figure 7). For phase I, II or III clinical trials, which do not generally include genetic data (which would make the set‐up of the protocol much more cumbersome and slow), e‐CE could be used for parallel recruitment. Furthermore, e‐CE could also be used for the pre‐recruitment of future participants at the time of creation of the protocol, making it possible to obtain more accurate evaluations of the real number of participants meeting the inclusion criteria. In any case, these individuals would also be invited to share their genetic data, to maximize sequencing efficiency. The financial surplus generated by this exchange would be used as explained in the previous figure.

**Figure 8:**
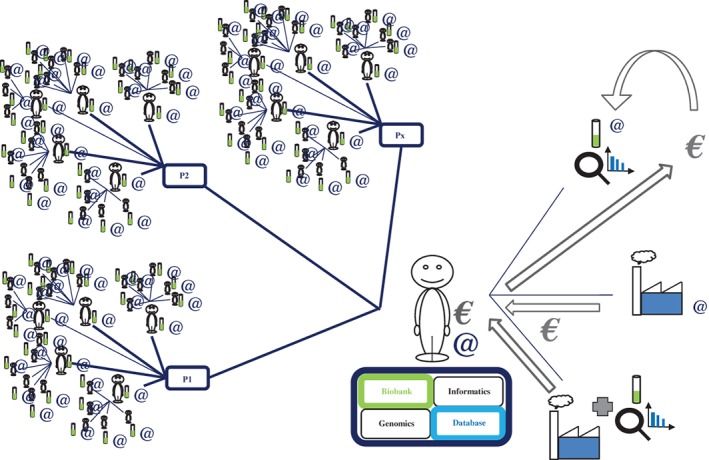
Modeling of interactions between participants, doctors and researchers, plus external researchers, companies and various sponsors. If other sponsors develop studies based on our e‐CE model (genetic data associated with dynamic questionnaires), they can organize themselves to collaborate for one or several questionnaires or to pose their questions to the other cohorts. The initial consent is unmodified and the participants can see the source of this question, like all the others. Thanks to the dynamic consent used in this cohort, it should also be possible to fuse cohorts or to switch sponsors, subject to consent from the participants [Colour figure can be viewed at http://wileyonlinelibrary.com]

Institutes, hospitals or research centers acting as study sponsors with this particular goal within the e‐CE model would derive considerable benefit from this system over collaborating with the different two‐sided markets, which are strictly illegal in France, under the legitimate and real argument of needing to access cohorts of several thousand connected individuals. For genetic studies, these organizations would be able to make use exclusively of e‐CE for all their investigators, thereby finding themselves in charge of large participatory cohorts (Figure 8). Furthermore, when a new laboratory wishing to perform human genetic studies wishes to join the cohort, a simple amendment to the protocol is required, to pose a new question to the participants of this large ready‐constituted cohort (network of doctors, computers, biobank, etc.). It would be suicidal not to set up such cohorts in France whilst 23andMe already has more than 10 million participants and French researchers and institutions have already used and published dozens of articles with this private company banned in France.

Furthermore, thanks to the use of dynamic consent,^12^, ^13^, ^14^, ^15^, ^16^, ^17^ if other sponsors develop a study based on a model similar to ours, they will be able to offer their participants the chance to collaborate with other cohorts for one or several questionnaires. Thus, even in the case of a single‐topic e‐CE, it remains of interest for both the participants and the sponsor to engage in such dynamic research. It is possible to collaborate just for one or several questionnaires, to increase independently the sizes of the different cohorts and/or even to fuse them, subject to the consent of the participants, to constitute a participatory “megacohort” (Figure 8), either definitively or temporarily. With a large volume of information of variable density, it should be possible to establish “real” big data or data mining based on transparent, united and fair French research. This would also provide French researchers with a solution enabling them to gain access to large cohorts rapidly without having to collaborate with the cohorts of companies banned in France.

Although purely theoretical at this stage, e‐CE respects not only French law, but also the new European general data protection rules (GDPR)‡‡‡‡ and follows the recent report on artificial intelligence from the French parliamentarian Cédric Villani,§§§§ and that of the French national ethics consultative committee (CCNE)***** concerning the production, usage, and ownership of personal data, particularly those of a genetic nature. The aspect of the ownership of genetic data deserves and will receive, much closer consideration, to improve the scientific and economic valorization of these data.^18^ In addition, e‐CE also respects the notion of developing a single identical protocol for genetic studies that in reality deal with multiple themes, rather than multiple protocols for single‐theme studies supported by a data warehouse (as currently recommended by the French data protection agency, the CNIL).

With such an organization and mode of functioning, e‐CE will facilitate the scientific use of genetic data in France, whilst respecting French and European laws and regulations. e‐CE will make it possible to perform research projects in human genetics more effectively and rapidly in France, whilst offering participants the possibility of direct, responsible and conscious involvement, validating the protocol de facto through this link of confidence.

### 1.3 Important issues and conclusion

In practice, of course, various issues of different natures must be taken into account for the effective implementation of such a system. First and foremost, there is a major scientific issue: improving the mapping of the human genome. It has become indispensable, to address this issue, to make it possible for all types of genetics studies authorized by the law to be performed within the same research protocol, rather than through data warehouses. Indeed, the use of a data warehouse in France requires the submission of a protocol to obtain authorization to study some of the data present in the warehouse, and this wastes time that could be more usefully spent on the protocol. e‐CE addresses this problem specifically. Rather than having to come up with a new research protocol with a specific theme and cohort each time, e‐CE makes it possible to study, within the same cohort, diverse topics, ranging from type 2 diabetes to face shape, for example. All this is important because, in the world of data, nothing must be lost; everything must be transformed, with the consent of the participant. Genetic data should have a broad possible scientific use, to make it easier to distinguish between genes linked to our health and genes with no apparent link to health, thereby allowing better mapping of the human genome.

This scientific issue gives rise to a number of important technical, financial and economic considerations. Indeed, such a project requires diverse technical means for the storage and communication of a large volume of biological samples and information between different people: the storage of samples, a computer server, computers, computer tablets, smartphones, internet networks, transport networks, without forgetting the technical means for applying the safety and quality standards in force to biological samples and genetic data. Fears about the time spent recruiting patients would result in the evaluation of fewer patients per day. Prior information, via an advertisement, in parallel with a doctor dedicated to this process, would solve this problem. In any case, at this stage, the many technical means required will require relatively high levels of funding, most of which will need to be found by the protocol.

However, we should not forget that a cohort of 10 million participants (ie, 23&Me) is currently considered to be worth 2 billion dollars, automatically leading to investment. Thus, in addition to possible public funding, the various partnerships envisaged with private companies should make it possible to cover these costs (Figures 5, 6 and 7) and, at the same time and in the same space, the development of e‐CE. Along the same lines, e‐CE does not rule out financial and scientific collaboration with foreign two‐sided markets, within the constraints of European and French standards. In this case, France would retain control over French genetic data, again with the express consent of the participants. Furthemore, we believe that the generalization of e‐CE would maintain and increase the quality of care without overloading the health system, because e‐CE is simply a way of optimizing current and future RIPHs within our health system. Furthermore, in the generalization of e‐CE, to prevent the cohort becoming enriched in disease‐prone individuals, we recommend the enrollment of all the individuals accompanying the patients, their helpers or spouses in the studies. This should make it possible to obtain cohorts including reasonably healthy people with a better distribution of ancestries and genealogies.

In addition to these scientific, technical, financial and economic issues, we must consider another set of issues that is just as important: ethical issues. Indeed, France appears to be opting for the continuation of a restrictive legal and moral framework concerning the production and use of genetic data.††††† This is not necessarily a bad choice in itself, as it makes it possible to prevent two‐sided markets from obtaining a monopoly on the production of genetic data in France.^9^ However, the law is not applied strictly, forcing researchers who wish to work on large cohorts to supply their questionnaires free‐of‐charge in return for a tiny amount of data, whilst these companies have direct access to the DNA of these participants. It is for this reason that such a protocol must be managed. e‐CE will meet this need by having a scientific and ethics committee composed of doctors, biologists, philosophers, bioethicists, legal experts, sociologists and economists, who will evaluate the pertinence and necessity of the various amendments, new questions or additions of new investigating centers proposed to the ethics committee (CPP) and/or the CNIL. This committee will also select the research teams receiving funding and will take decisions concerning the delivery of research results within the cohort.

Another important issue is control over French data, to prevent an information drain and to ensure that we can learn to analyze these data *en masse*. Indeed, it is sad that the report on the revision of French bioethics law (N1572), which anticipates the massive arrival of these illegal and often medically false or incomplete genetic tests in France, recommends costly solutions that would be dangerous for our healthcare system. Indeed, this report proposes increasing the number of genetic counselors to analyze these poor‐quality results, to reassure those who have illegally had such tests performed. Now, if, through the e‐CE system, we were to deliver high‐quality information directly to the French people within RIPHs, there would no longer be any advantage to resorting to these companies, some of which charge for their services and do not respect the GDPR, because a medically validated predisposition identified by e‐CE would lead to free healthcare in France. However, it should be borne in mind that in France, as in other European countries, genetic discrimination is strictly prohibited, whereas the United States allows a number of exceptions to this rule.

Thus, the major ethical issues relating to what essentially amounts to the industrialization of genetic data production include, in particular, the issue of who owns the data. Indeed, over the course of a few decades, we have passed from the local and artisanal production of genetic data to a worldwide and standardized system of data production. This industrialization has made it possible to increase the amount of genetic data generated and to increase production quality, but it has also led to genetic data being seen as commercial goods. In the eyes of French law, genetic data are considered to be an element of the human body that belongs to the donor and not an asset belonging to an owner (French Civil Code Article 16‐1). In other words, in France, our bodies and genetic data are considered to belong to us, but we do not own them. They are not considered to be a commodity, and certainly not a commercial one. The problem in France is therefore as follows: How can we correctly industrialize the production of genetic data, to remain competitive, if only scientifically, without considering genetic data as a commodity? The response is as complex as the question, and the implementation of e‐CE could help us to find the answer.

In any case, even at this theoretical stage, it is clear that e‐CE offers a relatively simple and obvious methodology, with various indirect arguments of different natures in its favor. The MFG‐2025 should make use of a single cohort, such as e‐CE, rather than multiple small protocols with very restricted goals. However, only a real implementation of this system would make it possible to test its real efficacy, as proposed by the CCNE (expert opinion 129******), with the testing of genetic information collection from the general population, in particular. e‐CE would constitute a real French‐style “Genetics version 2.0,” essential for France to remain competitive, both scientifically and economically, in terms of genetic research, in the face of these new foreign two‐sided markets, which, every day, extend their influence a little further, thereby creating a continuous drain of our genetic data abroad, to the detriment of France.

## CONFLICT OF INTEREST

The authors are involved in promoting this new type of French cohort.

## Data Availability

The data that support this article are openly available.

## Footnotes

*
https://www.ccne-ethique.fr/fr/publications/donnees-massives-et-sante-etat-des-lieux-prospective-et-nouvelles-questions-ethiques

†
https://solidarites-sante.gouv.fr/systeme-de-sante-et-medico-social/recherche-et-innovation/france-genomique

‡
https://drees.solidarites-sante.gouv.fr/etudes-et-statistiques/acces-aux-donnees-de-sante/article/health-data-hub

§
https://www.23andme.com/

**
https://www.ancestry.com/dna/

††
https://www.myheritage.fr/

‡‡
Code civil (Civil Code) ‐ Article 16‐10

§§
Decree no. 2016‐1537 dated November 162 016 relating to research on humans

***
https://www.dmp.fr/?SEC-64

†††
https://www.snds.gouv.fr/SNDS/Accueil

‡‡‡
https://www.ameli.fr/l-assurance-maladie/statistiques-et-publications/sniiram/finalites-du-sniiram.php

§§§
https://www.fhf.fr/gestion-hospitaliere/pmsi.html

****
https://www.snds.gouv.fr/SNDS/Composantes-du-SNDS

††††
https://www.ncbi.nlm.nih.gov/clinvar/docs/acmg/

‡‡‡‡
https://www.cnil.fr/fr/reglement-europeen-protection-donnees

§§§§
http://m.enseignementsup-recherche.gouv.fr/cid128577/rapport-de-cedric-villani-donner-un-sens-a-l-intelligence-artificielle-ia.html

*****
http://www.ccne-ethique.fr/

†††††
http://www.assemblee-nationale.fr/15/rap-info/i1572.asp

******
https://www.ccne-ethique.fr/sites/default/files/avis_129_vf.pdf
